# Alcoholic Treatment of Tuberculosis

**Published:** 1858-02

**Authors:** Thomas Cushing

**Affiliations:** North East, Pa.


### ART. V.—*Alcoholic Treatment of Tuberculosis.* By THOMAS CUSHING, North East, Pa.

The researches of modern physiologists have led to the following conclusions with regard to the disease called tuberculosis, viz:

1. It is a blood disease.

2. The morbid condition of the blood, present in this disease, consists in an excess of albuminous, and a deficiency of fatty matters.

3. The cause or causes of this loss of balance must be looked after among those circumstances which affect the composition of the blood.

The healthy performance of the function of nutrition requires that the repair of the various tissues should at least be commensurate with the waste of the same tissues which is continually taking place; and inasmuch as this waste and repair are carried on through the medium of the blood, any disturbance of them must necessarily affect the composition of that fluid. If the elements of fat do not exist in sufficient quantity in the food taken, or if they do exist and are not properly elaborated by the digestive organs, there must, of course, result a deficiency of this substance in the blood—the waste continuing to take place at its normal rate.

If, on the other hand, the elimination from the organism of adipose tissue takes place at an abnormally rapid rate—the supply from without continuing the same, that will occasion a deficiency of this substance in the circulating fluid.

It is not my purpose to enter into a discussion of the question whether tuberculosis is caused by one or the other or both of these deviations from a healthy performance of the function of nutrition, but to discuss briefly the therapeutical action, the modus operandi of a medicinal agent, the efficacy of which in the treatment of this affection late experience has amply demonstrated,—I mean alcohol.*

I propose first to speak of the influence which alcohol exerts upon the organism in health, and afterwards to show that its beneficial effect in the disease under consideration is due to a precisely similar action.

And here I will remark, that I am in no way disposed to undervalue the other remedial means which have been resorted to of late in the treatment of this affection, but rather to insist upon their employment in conjunction with this agent.

Let us now inquire what effect alcohol has upon the system of a person in health. Its first and most obvious effect is that of a stimulant to the brain and nervous system. By continuing the use of a given quantity daily, however, this effect is lost, or rather the brain and nervous system become so habituated to it, that under the influence of the accustomed quantity, they possess only their ordinary activity. If the quantity taken is sufficiently large, there is a marked diminution in the quantity of food consumed; in many cases not more than one-half or even one-fourth of the usual amount being taken.

Notwithstanding this diminution in the quantity of food, there is a steady and more or less rapid increase in the quantity of fat in the various tissues where it is ordinarily deposited.

Under these circumstances, one or more of the three following propositions must be true, viz:

1. The effect of the alcohol upon the digestive organs, is such that a larger proportion of alimentary matter is by them converted into fat.

2. The alcohol itself is converted into fat.

3. It prevents the elimination from the system of the fat furnished from the food.

With regard to the first proposition, I conceive that it can be true to a limited extent only; or at any rate, not to an extent sufficient to account for the phenomena observed in such cases.

It is a well-known fact that in many of these cases, we not only have no evidence of improvement in the action of the digestive organs, beyond the fact—if that is an evidence—of the increase in the quantity of fat in the system, but we have direct evidence of an imperfect performance of the function of digestion, in the existence of those symptoms which are characteristic of indigestion under all circumstances.

It may be said, that though there exist evidences of derangement in the performance of this function, yet the result of this very derangement may be the conversion of a larger than usual proportion of the food into fat.

To this, we answer, that we see the same evidences of deranged digestion in those who take no alcohol, and the consequence is a diminution instead of an increase, in the quantity of adipose matter.

As to the second proposition, I have only to say that the conversion of any substance into fat, is a process that takes place in the digestive organs alone; and that, as alcohol is not digested but absorbed and taken directly into the circulation, this metamorphosis of it cannot take place.

If, then, it is evident that the first two propositions are false, or only true to a limited extent, and not sufficient to account for the phenomena observed, that alone would be sufficient to prove the third true, and here I might rest; but in addition to this negative evidence of its truth, I propose to adduce some positive proof that it is in this way that alcohol produces the effect spoken of.

Dr. T. K. Chambers, in his work on "digestion and its derangements," gives the results of a series of experiments performed by Dr. Böcker, of Bonn, upon himself, for the purpose of ascertaining the effect of different agents—and among them alcohol—upon the nutrition of the organism. That these experiments might show a correct result, Dr. B. first ascertained the precise quantity of food and drink necessary to "exactly cover the outgoings of the organism," and during the continuance of the experiments, took just this quantity under the same circumstances as before.

The experiments with each substance extend over a period of seven days.

The results of these experiments, in the case of alcohol, are thus stated by Dr. Chambers:

"1. Alcohol diminishes the excretion both of the solid and fluid constituents of the urine.

"2. Alcohol does not increase to any appreciable extent the cutaneous perspiration.

"3. Alcohol does not augment the fœcal excretion.

"4. Alcohol diminishes not only the absolute quantity of carbonic acid exhaled by the lungs, but also the relative proportion of it in the products of respiration, though it increases the frequency of the respiratory movements.

"5. Alcohol does not affect the excretion of water from the lungs to any important extent."

The Dr.'s tables show that the diminution of urine, carbonic acid, and water, were as follows: urine $\frac{4}{10}$ of the whole average quantity of its fluid and solid constituents.

Carbonic acid $\frac{1}{5}$ less than the ordinary quantity, water $\frac{1}{135}$ of the whole—an inconsiderable amount.

The results of these experiments, which I think have been verified by others, show conclusively that alcohol affects the organism in the way spoken of in the third proposition, viz., by arresting the metamorphosis of the tissues, and preventing the excretion or elimination of the materials which compose them.

I come now to speak of the effect of alcohol upon persons affected with tuberculosis:

The result of my own observations is, that patients with this disease will bear larger quantities of this agent, without feeling any of its ordinary effects upon the brain and nervous system than in health; and this I find is the result of the observation of others.

If the view which I take of the pathological condition in this disease is correct, and if the therapeutical action of alcohol is what the experiments alluded to seem to show, the fact of larger quantities being tolerated by tuberculous patients is easily explained. If not, I cannot account for it.

It is now generally admitted by physiologists, that the carbonic acid which is exhaled from the lungs of a person in health, is the result of a combination or union in the capillaries, of the carbonaceous matter of the tissues with the oxygen of the inspired air,—and that in this way the elimination of a large portion of this carbonaceous or fatty matter is accomplished. If a given quantity of alcohol is introduced into the circulation, a portion of it is eliminated without change, through the lungs.

During its circulation through the capillaries, however, it is brought in contact with the oxygen which the red discs of the blood have brought from the lungs, and combustion takes place,—a portion of the oxygen of the arterial blood uniting with the carbon of a portion of the alcohol to form carbonic acid, which is taken back to the lungs by the discs of the venous blood and exhaled. The portion of alcohol thus consumed in the capillaries of a healthy person is comparatively small, because the oxygen of the arterial blood finds just without the walls of the capillaries, the fat which has been elaborated from the food and deposited there, and with the carbon of this fat, it also combines.

The alcohol which is thus slowly decomposed in the capillaries, or exhaled through the pulmonary mucous membrane, is kept longer in the circulation than would be the case were it more rapidly consumed; and by being brought in contact with the entire surface of the nervous system and brain, is enabled to exert its specific influence upon them.

If, however, the same quantity of alcohol is introduced into the circulation of a person in whom there is a deficiency of fat or carbonaceous matter, the union of its carbon with oxygen in the capillaries takes place more rapidly, and less of the alcohol is left to exercise its stimulating influence. This I believe to be the true reason why alcohol exerts less of a stimulating influence upon persons affected with tuberculosis than upon others in health, and this I believe to be the methodus medendi of alcohol in tuberculosis. According to these views, it is to be regarded not as a stimulant that exerts a temporary influence and leaves the system to suffer from the consequent reäction, nor as a vital stimulant, capable of maintaining the function of nutritition by furnishing the materials for repairing the waste of the tissues, but as an "accessory food," which, by checking this waste and economizing the resources of nature, enables the debilitated system to regain its vigor. If it be objected to the use of alcohol under these circumstances, that there is danger of its engendering habits of intemperance, I have only to say in reply, that I am not one of those who would pluck out a man's tongue through fear that "the sacred faculty of speech" might be "turned to impious use."

